# Association between polymorphisms in the promoter region of T cell immunoglobulin and mucin domain-3 and myasthenia gravis-associated thymoma

**DOI:** 10.3892/ol.2015.2845

**Published:** 2015-01-05

**Authors:** GUOWU XU, KAI ZHENG, XING LU, JINXIANG WANG, YANFEN CHAI, JUNYI WANG

**Affiliations:** 1 Department of Emergency, Tianjin Medical University General Hospital, Tianjin 300052, P.R. China; 2 Department of Cardiothoracic Surgery, Tianjin Medical University General Hospital, Tianjin 300052, P.R. China; 3 Department of Intensive Care Unit, Tianjin Third Central Hospital, Tianjin 300170, P.R. China

**Keywords:** thymoma, myasthenia gravis, T-cell immunoglobulin and mucin domain-3, gene polymorphism

## Abstract

Thymoma is a type of benign or low-grade malignant tumor, occurring on the thymic epithelium. Patients with thymoma may also suffer from myasthenia gravis (MG), presenting MG-associated thymoma. T cell immunoglobulin and mucin domain-3 (Tim-3), a subtype of the Tim protein family, may be an important immune regulatory and pivotal molecule associated with tumor development. In order to understand the etiology and pathogenesis of MG-associated thymoma in the Han population of North China, the present study investigated the association between a polymorphism on the −574 locus in the promoter of Tim-3 and the risk of MG-associated thymoma in the Han Chinese population. In total, 116 patients with thymoma and MG were enrolled into the MG-associated thymoma group, while 124 patients with thymoma, but without MG, were enrolled into the non-MG-associated thymoma group. Examinations were conducted to reach a definite diagnosis of thymoma and MG and rule out other autoimmune diseases. Allele-specific polymerase chain reaction (AS-PCR) was performed to determine the polymorphism on the −574 locus of Tim-3 in all the subjects. PCR products were randomly selected for sequencing. Statistically significant differences were detected between the distribution frequencies of the GT+TT genotype and T allele on the −574 locus of the MG-associated thymoma group (31.03 vs. 12.90%, respectively; χ^2^=11.609, P=0.001) and the non-MG-associated thymoma group (15.52 vs. 6.45%, respectively; χ^2^=10.198, P=0.001). In conclusion, the present study indicated that an association may exist between the polymorphism of the −574 locus in the Tim-3 promoter and MG-associated thymoma.

## Introduction

Thymoma is a type of benign or low-grade malignant tumor, developed on the thymic epithelium, and the most frequent anterior mediastinal neoplasm in adults; however, its overall incidence is only 0.13 per 100,000 individuals/year according to data from the Surveillance, Epidemiology and End Results database (1–3). Due to the low incidence of this neoplasm, its etiology and pathogenesis remain unknown. In addition, epidemiological data have revealed that 20–25% of patients with thymomas also suffer from myasthenia gravis (MG; hereafter referred to as MG-associated thymoma), whereas 10–20% of myasthenic patients suffer from thymomas (4). MG, is an autoimmune disease of unclear etiology, results in even more complex etiology and pathogenesis of MG-associated thymoma. The ratio of patients with thymoma and MG is higher than that of any ofther autoimmune disease. Pivotal markers and cross-linked molecules of thymoma and MG have been the aim of several previous studies (5,6).

Thymus is a primary organ and the initial site for the development of the T cell immunological function (7). The development of thymoma is frequently accompanied by a rich infiltrate of T cells (3). When released into the circulation, these abnormally conditioned T cells are hypothesized to be responsible for the autoimmune conditions that often accompany thymoma, particularly MG (3). MG is a known prototypical CD4^+^ T cell-dependent autoimmune disease. Various subsets of T helper (Th) cells, including Th1, Th2 and Th17, and regulatory T cells (Treg) have been suggested to be involved in the pathogenesis of MG (8). In addition, the imbalance of different Th cell subsets is important in the progression of the disease. Thymoma and MG are multifactorial and noninherited diseases, with an established genetic predisposition. Numerous studies have demonstrated that certain human leukocyte antigen (HLA) and non-HLA genes, including HLA-death receptor 3, PTPN22 and CTLA4, present an evident association with MG (9). However, a clear correlation of particular genes/molecules with MG-associated thymoma cannot be gained from the limited present research (10).

T cell immunoglobulin and mucin domain-3 (Tim-3) is a subtype of the Tim protein family, which was initially identified in 2002, and is selectively expressed on Th1 cells, but not on Th2 cells (11). Tim-3 has been demonstrated to negatively regulate Th1 response and induce immune tolerance through the Tim-1/galectin-9 signaling pathway in autoimmune diseases (12). Furthermore, suppression of Tim-3 expression has been revealed to enhance the pathological severity of experimental autoimmune encephalomyelitis (EAE) (13), while Tim-3-deficient mice have been found to be refractory to the induction of immune tolerance in EAE (14). Besides the important role of Tim-3 in autoimmune diseases, a recent study has indicated that Tim-3 is also a molecular switch for tumor escape from innate immunity (15). Previous studies have further identified Tim-3 expression on exhausted T cells in human tumors (16,17) and preclinical tumor models (18,19). The expression of Tim-3 is significantly increased on T cells in tumor-infiltrated tissues and on tumor-infiltrating lymphocytes in peripheral lymphoid tissues or the blood of tumor patients, indicating that Tim-3 may be upregulated in a tumor-derived environment (17,18). Therefore, the present study hypothesized that Tim-3 may be a potentially pivotal molecule in autoimmune disease-associated tumors, such as MG-associated thymoma.

Tim-3 polymorphism has been demonstrated to alter the interaction between Tim-3 and its ligand, thereby affecting the process that results in certain immune diseases (19,20) and being actively involved in the pathogenesis of tumors (21). Considering the aforementioned findings, Tim-3 appears to be an important regulatory molecule that plays a critical role in MG-associated thymoma, which may be triggered mainly by the deviation of Th cell subtypes. However, to the best of our knowledge, Tim-3 polymorphism and its association with MG-associated thymoma in the Han Chinese population of North China have not been evaluated. The aim of the present study was to investigate the polymorphism of the −574 locus in the promoter of Tim-3 and its association with MG-associated thymoma in the Han Chinese population of North China.

## Materials and methods

### Study approval

The experiments of this study were approved by the Ethics Committee of the Tianjin Medical University General Hospital (Tianjin, China). Written informed consent was obtained from all the patients prior to participation in the study. All the subjects were from the Han population of North China, since the study was performed at Tianjin, China.

### Patients and DNA samples

In total, 116 patients with thymoma and MG were enrolled from the Department of Cardiothoracic Surgery of the Tianjin Medical University General Hospital, comprising the study group (MG-associated thymoma group). In addition, 124 patients with thymoma, but without MG, were randomly selected from the Department of Cardiothoracic Surgery of the Tianjin Medical University General Hospital, forming the control group (non-MG-associated thymoma group). All the subjects were subjected to a comprehensive examination in order to obtain a definite diagnosis of thymoma and MG and exclude other autoimmune diseases. For the diagnosis of thymoma, the results of chest X-ray, computed tomography and magnetic resonance imaging, as well as surgical and pathological findings, were considered. The diagnostic criteria of MG included myasthenic manifestation, neostigmine test, repetitive nerve stimulation test and serum autoantibody detection. All the patients included in this study were genetically unrelated individuals of the Han Chinese population. Genomic DNA was extracted from the white blood cells of each sample using a DNA isolation kit (GK1072, Generay Biotech Co, Ltd., Shanghai, China), according to the manufacturer’s instructions. The extracted genomic DNA was dissolved in sterile double-distilled water. Subsequently, the concentrations and absorbance ratios at 260 nm/280 nm (A260/A280) of the DNA solutions were measured using a nucleic acid spectrometer (ScanDrop 200; Analytik Jena AG, Jena, Germany). DNA samples with A260/A280 ratios ranging between 1.7 and 2.0 were selected as polymerase chain reaction (PCR) templates and stored at −80°C.

### -574 locus genotyping

Allele-specific PCR (AS-PCR) was used to investigate the −574 locus and three primers were designed using Primer 5.0 software (Premier Biosoft, Palo Alto, CA, USA), according to the flanking sequence (GenBank no. NM_032782). The primer sequences were as follows: Forward 1 (F1), 5′-GGCTTATGCTGGGAGTTGCT-3′; forward 2 (F2), 5′-GGCTTATGCTGGGAGTTGCG-3′; reverse for F1 and F2 (R), 5′-GGTGTCTGATTGCCAGTGATTC-3′. The F1 and R primers were used to amplify T allele fragments, whereas F2 and R primers were used to amplify G allele fragments. All the amplified fragments were 539 bp long and each sample was subjected to AS-PCR with F1/R and F2/R. The reaction mixture was as follows: 2.5 μl 10× PCR buffer; 1.5 μl deoxyribonucleotide triphosphate (2.5 mmol/l each); 1 μl forward primer (5 μmol/l); 1 μl reverse primer (5 μmol/l); 1.0 μl DNA template; and 0.5 μl Taq DNA polymerase (2.5 U/μl) (Beijing AuGCT DNA-Syn Biotechnology Co., Ltd., Beijing, China). Double-distilled water was added to obtain a final reaction volume of 25 μl and, subsequently, touchdown PCR was performed to amplify the target amplicons. The reaction mixture was heated in a Thermo Cycler (Mastercycler 5333; Eppendorf AG, Hamburg, Germany) under the following conditions: 95°C for 5 min; 94°C for 30 sec; progressive lowering of the annealing temperature by 1°C every three 30 sec annealing cycles (between 65°C and 56°C); extension at 65°C for 35 sec, followed by 30 cycles at 72°C for 35 sec; final extension at 72°C for 10 min. The amplified products were subjected to 1.8% agarose gel electrophoresis and visualized using ethidium bromide staining, followed by capturing images using FireReader software (XS D5626M Auto + FC26WL + Uviband; UVItec Ltd., Cambridge, UK).

### DNA sequencing

Several PCR products were randomly selected and purified using a PCR purification kit (DK004-01B; NovoProtein Biotechnology Co., Ltd., Shanghai, China). Sequencing of the purified products was performed by Beijing AuGCT DNA-SYN Biotechnology Co., Ltd. (Beijing, China).

### Statistical analyses

Statistical analysis was performed using the SPSS 17.0 software package (SPSS, Inc., Chicago, IL, USA). χ^2^ test was performed to compare the allele frequencies of each group, while the Hardy-Weinberg equilibrium test was conducted to investigate the demographic representation of the study and control groups. The relative risk was presented as the odds ratio (OR) and its 95% confidential interval (CI). P<0.05 was considered to indicate a statistically significant difference.

## Results

### General characteristics

The ages of the MG-associated thymoma patients were 18–62 years with a mean age of 45.6±4.5 years, while the ages of the non-MG-associated thymoma patients were 20–63 years with a mean age of 46.4±4.3 years. No statistically significant difference was observed between the mean ages of the two patient groups (P>0.05). In addition, the Hardy-Weinberg equilibrium test results for the −574 locus revealed no statistically significant differences between the study and control groups (P>0.05), which indicated that the two groups were in Hardy-Weinberg equilibrium and demographically representative.

### -574 locus

**Figure 1:**
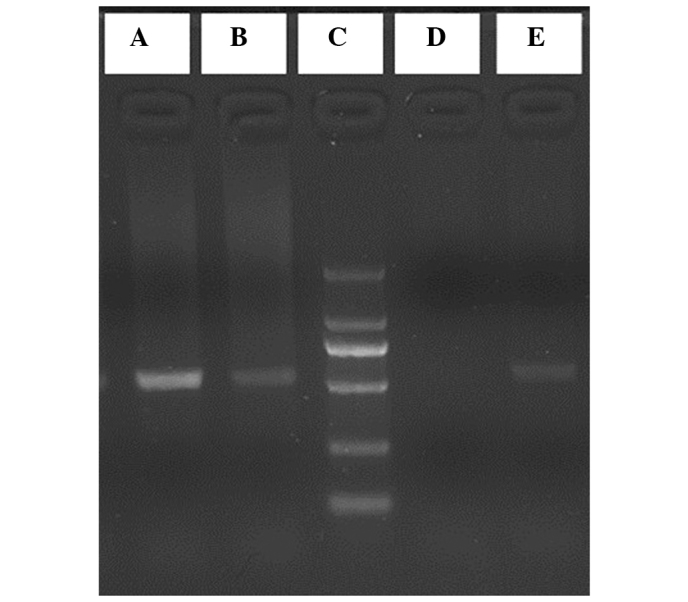
Electrophoretogram of −574 locus in the Tim-3 gene. A and B represent the TG genotype; C represents the DNA marker with 2000, 1000, 750, 500, 250 and 100 bp; D and E represent the GG genotype. Tim-3, T cell immunoglobulin and mucin domain-3;

**Figure 2:**
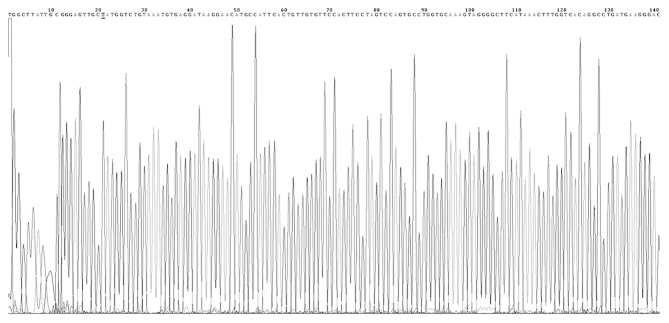
Sequence diagram of T allele amplified product at the −574 locus of the Tim-3 promoter region. T allele is underlined.

**Figure 3:**
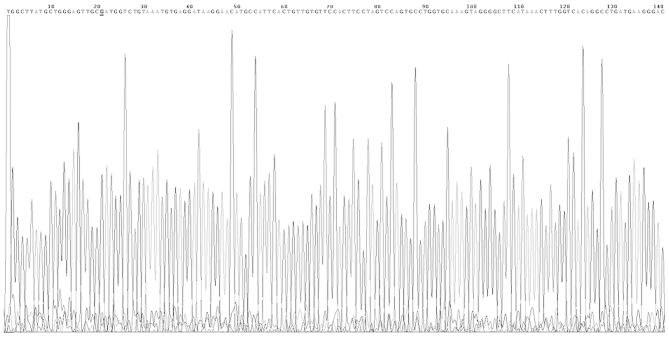
Sequence diagram of G allele amplified product at the −574 locus of the Tim-3 promoter region. G allele is underlined.

At the −574 locus, the G and T alleles were defined as wild-type and mutant alleles, respectively. Following agarose gel electrophoresis, the GG genotypes were defined as wild GG homozygotes, the TT genotypes were defined as mutant TT homozygotes and the GT genotypes were defined as GT heterozygotes (Fig. 1). In the present study, the GG genotype was identified in 80 MG-associated thymoma patients, while the GT genotype was identified in the remaining 36 MG-associated thymoma patients. By contrast, the GG genotype was detected in 108 non-MG-associated thymoma patients, while the GT genotype was detected in the remaining 16 patients of the group. The TT genotype was not identified in the study or control patients (Figs. 2 and 3).

### Genotypes and frequencies

At the −574 locus of Tim-3 in the MG-associated group, the GG and (GT+TT) genotypes presented frequencies of 68.97 and 31.03%, respectively. By contrast, 87.10% of the control subjects possessed the GG genotype and the remaining 12.90% possessed the (GT+TT) genotype. A statistically significant difference was observed between the two groups (χ^2^=11.609, P=0.001). The frequency of the GT+TT genotype on the −574 locus of the MG-associated thymoma group was found to be statistically different compared with the control group (P=0.001, OR=0.329; Table I). In addition, the frequencies of T allele on the −574 loci of the two groups were statistically different (P=0.001, OR=0.375; Table II).

## Discussion

Tim-3, a surface molecule on T cells, is important in immune regulatory functions and presents a strong correlation with various tumor types. In humans, Tim-3 is located on chromosome 5q33.2 and comprises 301 amino acids, including elementary structural domains, such as the signal domain, mucin-like structural domain, transmembrane zone and cytomere domain of the phosphorylation site (13–17,20). In the immunoglobulin V structural domain, two antiparallel β fragments and a metal ion ligand binding site function together as the ligand-binding site of Tim-3 (14,23).

Evidence indicates that Tim-3 is a negative regulator of T cell responses and is involved in the modulation of autoimmune diseases (20). Identification of galectin-9 as a ligand of Tim-3 may induce the death of Th1 cells and downregulate Th1 responses (12,23). A previous study demonstrated that *in vivo* treatment with Tim-3 monoclonal antibodies during the induction of EAE, which is a mouse model of multiple sclerosis (MS), accelerated disease progression (11). In human patients with MS, T cell clones derived from the cerebrospinal fluid expressed lower levels of Tim-3 compared with those from healthy control subjects (24). A study on a mouse model of autoimmune diseases has indicated that inhibition of the interaction between Tim-3 and its ligand may dramatically aggravate the manifestation of autoimmune diseases (25). Furthermore, the distribution frequencies of +4259 T/G in Tim-3 in patients with rheumatoid arthritis have been demonstrated to be statistically different compared with healthy individuals in the Han and Hui Chinese populations (26). Therefore, the present study hypothesized that Tim-3 may be a protective factor of autoimmune diseases (it may protect patients from suffering from autoimmune diseases or decrease the possibility of suffering from autoimmune diseases), while Tim-3 polymorphisms may be closely associated with autoimmune diseases.

The role of Tim-3 in tumor tissues has been investigated in numerous studies. Piao *et al* (27) identified that Tim-3 expression was higher in prostate cancer tissues compared with the adjacent benign tissues. In addition, Cao *et al* (28) revealed that Tim-3 expression in cervical cancer promoted tumor metastasis. Furthermore, a number of studies identified that the distribution frequencies of +4259 T/G in Tim-3 in patients with pancreatic cancer or renal cell carcinoma were statistically different compared with healthy individuals (29,30). A recent study also demonstrated that, following tumor-associated expression of the receptor Tim-3 by dendritic cells, Tim-3 inhibited the antitumor efficacy of DNA vaccines and chemotherapy (31). Therefore, Tim-3 may be a risk factor of tumorigenesis, while Tim-3 polymorphisms may be associated with cancer.

In the present study, polymorphisms at the −574 locus of Tim-3 were investigated. Statistical analysis revealed that, in the MG-associated and non-MG-associated thymoma groups, the mutant-type homozygote TT frequencies were zero. Thus, the mutant-type homozygote TT and heterozygote GT phenotypes were merged and reanalyzed using the χ^2^ test. The results detected a statistically significant difference in the distribution frequencies of the GT+TT genotype between the two groups (Table I). In addition, the distribution frequencies of T allele on the −574 locus were significantly different between the two groups (Table II). These findings indicated an association between the −574 locus polymorphism and MG-associated thymoma in the Han population of North China. Furthermore, the OR values were found to be 0.329 and 0.375 for the distribution frequencies of the GT+TT genotype and T allele, respectively, on the −574 locus between the two groups. Based on the results of the aforementioned studies, Tim-3 was hypothesized to be a protective factor in autoimmune diseases and a risk factor of tumorigenesis; however, to date, the association between tumorigenesis and autoimmune diseases remains unclear. Thus, the present study investigated the correlation between Tim-3 and MG-associated thymoma, which is a tumor commonly associated with autoimmune diseases. In conclusion, Tim-3 was found to be a protective factor in MG-associated thymoma, indicating that thymoma is affected by MG. Therefore, future research on thymoma should investigate the possible association with MG.

## Figures and Tables

**Table I:** Distribution of -574 locus of Tim-3 genotypes of the studied population (%).

Genotype	Study group (n=116), n (%)	Control group (n=124), n (%)	χ^2^-value	P-value	OR	95% CI
GG	80 (68.97)	108 (87.10)	11.609	0.001	0.329	0.171–0.634
GT+TT	36+0 (31.03)	16+0 (12.90)				

Tim-3, T cell immunoglobulin and mucin domain-3; OR, odds ratio; CI, confidence interval.

**Table II:** Distribution of different alleles on the -574 locus.

Alleles	Study group (n=116), n (%)	Control group (n=124), n (%)	χ^2^-value	P-value	OR	95% CI
G	196 (84.48)	232 (93.55)	10.198	0.001	0.375	0.202–0.697
T	36 (15.52)	16 (6.45)				

## Abbreviations

AS-PCR: allele-specific polymerase chain reaction
CI: confidence interval
EAE: experimental autoimmune encephalomyelitis
MG: myasthenia gravis
MS: multiple sclerosis
OR: odds ratio
Tim: T-cell immunoglobulin and mucin domain

## References

[1] Priola AM, Priola SM (2014). Imaging of thymus in myasthenia gravis: from thymic hyperplasia to thymic tumor. Clin Radiol.

[2] Shapiro M, Korst RJ (2014). Surgical approaches for stage IVA thymic epithelial tumors. Front Oncol.

[3] Engels EA (2010). Epidemiology of thymoma and associated malignancies. J Thorac Oncol.

[4] Lucchi M, Ricciard R, Melfi F (2009). Association of thymoma and myasthenia gravis: oncological and neurological results of the surgical treatment. Eur J Cardiothorac Surg.

[5] Yu L, Zhang XJ, Ma S (2012). Different characteristics of thymomas with and without myasthenia gravis. Ann Surg Oncol.

[6] Zheng K, Xu G, Lu X (2014). Expression and polymorphisms of T cell immunoglobulin domain and mucin domain protein-1 in thymoma with or without myasthenia gravis. Oncol Lett.

[7] Pearse G (2006). Normal structure, function and histology of the thymus. Toxicol Pathol.

[8] Wang Z, Wang W, Chen Y, Wei D (2012). T helper type 17 cells expand in patients with myasthenia-associated thymoma. Scand J Immunol.

[9] Zagoriti Z, Kambouris ME, Patrinos GP (2013). Recent advance in genetic predisposition of myasthenia gravis. Biomed Res Int.

[10] Zheng K, Zhang J, Guo Y, Zhang P (2013). Expression and clinical significance of protein tyrosine phosphatase nonreceptor 22 in resected thymoma. Clin Lab.

[11] Monney L, Sabatos CA, Gaglia JL (2002). Th1-specific cell surface protein Tim-3 regulates macrophage activation and severity of an autoimmune disease. Nature.

[12] Zhu C, Anderson AC, Schubart A (2005). The Tim-3 ligand galectin-9 negatively regulates T helper type 1 immunity. Nat Immunol.

[13] Anderson AC, Anderson DE (2006). TIM-3 in autoimmunity. Curr Opin Immunol.

[14] Sabatos CA, Chakravarti S, Cha E (2003). Interaction of Tim-3 and Tim-3 ligand regulates T helper type 1 responses and induction of peripheral tolerance. Nat Immunol.

[15] Mattei F, Schiavoni G (2013). TIM-3 as a molecular switch for tumor escape from innate immunity. Front Immunol.

[16] Fourcade J, Sun Z, Benallaoua M (2010). Upregulation of Tim-3 and PD-1 expression is associated with tumor antigen-specific CD8^+^ T cell dysfunction in melanoma patients. J Exp Med.

[17] Baitsch L, Baumgaertner P, Devêvre E (2011). Exhaustion of tumor-specific CD8^+^ T cells in metastases from melanoma patients. J Clin Inverst.

[18] Sakuishi K, Apetoh L, Sullivan JM (2010). Targeting Tim-3 and PD-1 pathways to reverse T cell exhaustion and restore anti-tumor immunity. J Exp Med.

[19] Zhou Q, Munger ME, Veenstra RG (2011). Coexpression of Tim-3 and PD-1 identifies a CD8^+^ T-cell exhaustion phenotype in mice with disseminated acute myelogenous leukemia. Blood.

[20] Mclntire JJ, Umetsu SE, Macaubas C (2003). Immunology: hepatitis A virus link to atopic disease. Nature.

[21] Shen Y, Wang C, Hong D (2013). The relationship between polymorphisms in the promoter region of Tim-3 and unexplained recurrent spontaneous abortion in Han Chinese women. Reprod Biol Endocrinol.

[22] Shang Y, Li Z, Li H (2013). TIM-3 expression in human osteosarcoma: Correlation with the expression of epithelial-mesenchymal transition-specific biomarkers. Oncol Lett.

[23] Freeman GJ, Casasnovas JM, Umetsu DT, DeKruyff RH (2010). TIM genes: a family of cell surface phosphatidylserine receptors that regulate innate and adaptive immunity. Immunol Rev.

[24] Koguchi K, Anderson DE, Yang L (2006). Dysregulated T cell expression of TIM3 in multiple sclerosis. J Exp Med.

[25] Meyers JH, Sabatos CA, Chakravarti S, Kuchroo VK (2005). The TIM gene family regulates autoimmune and allergic diseases. Trends Mol Med.

[26] Xu J, Yang Y, Liu X, Wang Y (2011). The -1541C>T and +4259G>T of TIM-3 polymorphisms are associated with rheumatoid arthritis susceptibility in a Chinese Hui population. Int J Immunogenet.

[27] Piao YR, Piao LZ, Zhu LH (2013). Prognostic value of T cell immunoglobulin mucin-3 in prostate cancer. Asian Pac J Cancer Prev.

[28] Cao Y, Zhou X, Huang X (2013). Tim-3 expression in cervical cancer promotes tumor metastasis. PLoS One.

[29] Cai C, Wang L, Wu Z (2012). T-cell immunoglobulin- and mucin-domain-containing molecule 3 gene polymorphisms and renal cell carcinoma. DNA Cell Biol.

[30] Tong D, Zhou Y, Chen W (2012). T cell immunoglobulin- and mucin-domain-containing molecule 3 gene polymorphisms and susceptibility to pancreatic cancer. Mol Biol Rep.

[31] Tang D, Lotze MT (2012). Tumor immunity times out: TIM-3 and HMGB1. Nat Immunol.

